# Novel Intranasal Replication-Deficient NS1ΔC Flu Vaccine Confers Protection from Divergent Influenza A and B Viruses in Mice

**DOI:** 10.3390/vaccines14010043

**Published:** 2025-12-30

**Authors:** Daria Shamakova, Marina A. Shuklina, Nikita Yolshin, Ekaterina Romanovskaya-Romanko, Anna-Polina Shurygina, Kira Kudrya, Arman Muzhikyan, Mariia V. Sergeeva, Marina Stukova

**Affiliations:** Smorodintsev Research Institute of Influenza, Ministry of Health of the Russian Federation, 197022 St. Petersburg, Russianikita.yolshin@gmail.com (N.Y.); romromka@yandex.ru (E.R.-R.);

**Keywords:** universal influenza vaccine, influenza A virus, influenza B virus, NS1 truncation, replication-deficient vaccine, intranasal administration, cross-protection, immune response

## Abstract

**Background/Objectives**: The current strategy for seasonal influenza prophylaxis relies on updating the vaccine components annually to account for the rapid antigenic drift of viruses and the low cross-protective efficacy of available vaccines. Mutant influenza viruses with truncated or deleted NS1 protein are known to stimulate cross-specific T-cell immune response and provide protection against heterosubtypic influenza A and B viruses. **Methods**: We generated NS1ΔC influenza A and B viruses with C-terminal NS1 deletions by reverse genetics. In a mouse model, we assessed the safety and immunogenicity of the B/Lee/NS1ΔC strain upon intranasal administration, as well as the mechanism of its cross-protective efficacy against sublethal B/Victoria and B/Yamagata challenges. We then investigated the potential of the intranasal Flu/NS1ΔC vaccine–a trivalent formulation of NS1ΔC A/H1N1, A/H3N2, and B influenza viruses–to protect mice from lethal influenza infection with homologous, heterologous, and antigenically drifted influenza A and B viruses. **Results**: Intranasal immunization with the B/Lee/NS1ΔC strain was safe in mice. It activated cross-specific T-cell responses in the lungs and protected animals against heterologous challenge by reducing viral load, inflammation, and lung pathology. Immunization with the trivalent Flu/NS1ΔC vaccine formulation improved survival and reduced weight loss and viral load upon challenge with A/H1N1pdm, A/H2N2, A/H5N1, and B/Victoria viruses. **Conclusions**: The trivalent intranasal Flu/NS1ΔC influenza vaccine is a promising tool to improve seasonal influenza protection and preparedness for an influenza pandemic.

## 1. Introduction

Influenza virus remains one of the major threats to public health, and vaccines continue to be the primary means of controlling it. However, frequent antigenic mutations in influenza viruses make it necessary to update the vaccine composition each season.

Currently available inactivated influenza vaccines (IIVs) induce neutralizing antibodies that bind predominantly to the highly variable globular head of haemagglutinin (HA). IIVs provide good protection against severe disease and mortality [1], but their effectiveness decreases if there is a mismatch between the vaccine strain and the circulating virus [2,3]. Unlike IIVs, live attenuated influenza vaccines (LAIVs) are administered intranasally, mimicking the natural route of influenza infection. LAIVs stimulate serum antibody production, accompanied by activation of the local immune response [4]. The vaccine virus strains replicate in the nasopharynx of immunized individuals, promoting the formation of resident memory T cells that provide long-lasting local immunity in the respiratory tract [5].

However, if the circulating virus is antigenically distinct, LAIV immunization becomes insufficient to elicit effective cross-protection [6]. One reason for the low immunogenicity of LAIV may be the expression of the fully functional NS1 protein that suppresses both innate and adaptive immune responses [7]. Therefore, removing the effector C-terminal domain by truncating the influenza NS1 protein could be another strategy to improve immunogenicity of an LAIV strain. Immunization with such mutant viruses enhances immune response in comparison to conventional IIVs and LAIVs [8,9] and provides broad cross-protection [10].

Mutant influenza B viruses with truncated NS1 are characterized by an attenuated phenotype and decreased replication in interferon-competent systems, which inversely correlates with the length of the NS1 protein [10,11,12,13]. The idea behind this study was to create an influenza B virus with NS1_1–183_ as a structural homolog of the influenza A NS1_1–124_ construct [14]. This achieves the optimal balance between preventing the virus from inhibiting the interferon system due to deletion of the NS1 C-terminal effector domain (NS1ΔC) and maintaining sufficient replication in chicken embryos for vaccine production. Here, we constructed the NS1ΔC influenza B virus and evaluated its immunogenicity and cross-protective efficacy in a mouse model. We then used three NS1ΔC influenza viruses, A/H1N1, A/H3N2, and B, to formulate the trivalent universal influenza vaccine Flu/NS1ΔC and tested its protective activity against heterologous and pre-pandemic influenza strains.

## 2. Materials and Methods

### 2.1. Recombinant Influenza Viruses with Truncated NS1

The recombinant influenza viruses with a truncated NS1 protein were obtained using reverse genetics and the pHW2000 plasmid [15,16]. Whole-length genome segments of the corresponding influenza A and B viruses were amplified and cloned into BsmBI/BsaI (New England Biolabs, MA, USA) restriction sites to generate plasmid sets encoding the eight viral genome segments. Viruses were rescued after transfection of a co-culture of MDCK cells (#CCL-34, ATCC, VA, USA) and HEK293FT cells (#R70007, Invitrogen, MA, USA). Transfection was performed in an overnight cell monolayer (1:1) using Lipofectamine^®^ LTX (Invitrogen, MA, USA) and 0.5 μg of each plasmid. Twenty-four hours after transfection, the culture and transfection medium DMEM/F12 with 10% FBS (Biolot, St Petersburg, Russia) was exchanged for the virus cultivation medium AlphaMEM with 1 μg/mL TPCK-trypsin (Sigma-Aldrich, MO, USA) and no serum. Rescued viruses were propagated and cloned in chicken embryos until their genetic and reproductive properties stabilized. Whole-genome virus sequences were obtained by NGS (MiSeq Illumina, CA, USA) following a previously developed protocol [17].

The recombinant A/PR8/NS1ΔC (H1N1) virus has been described earlier [18]. The recombinant A/Cambodia/PR8 6:2/NS1ΔC (H3N2) strain used in this study was a 6:2 reassortant based on the high-yield A/PR/8/1934 (H1N1) donor with a modified NS gene and the hemagglutinin (HA) and neuraminidase (NA) surface antigens from the A/Cambodia/e0826360/2020 (H3N2) virus. The NS gene modification involved truncating the NS1 protein after 124 amino acids, as described previously [19]. The recombinant prototype virus strain, B/Lee/NS1ΔC, represented the B/Lee/1940 virus with a modified NS gene resulting from spontaneous mutation, which produced the NS1 protein truncated after 176 amino acids.

The prototype trivalent Flu/NS1ΔC vaccine formulation contained A/PR8/NS1ΔC (H1N1), A/Cambodia/PR8 6:2/NS1ΔC (H3N2), and B/Lee/NS1ΔC viruses, each at a dose of 6.5 lg EID_50_/mL.

### 2.2. Wild-Type Influenza Viruses

Reference influenza viruses were obtained from the WHO Collaborating Centers for Reference and Research on Influenza as a part of the GISRS program for the National Influenza Centre at the Smorodintsev Research Institute of Influenza. Particularly, the B/Phuket/3073/2013, B/Washington/02/2019, and B/Malaysia/2506/2004 strains were provided by the Center for Disease Control and Prevention (Atlanta, GA, USA), and A/Cambodia/e0826360/2020 (H3N2) and B/Austria/1359417/2021 were obtained from the Francis Crick Institute, World Influenza Centre (London, UK). The ancestral B/Lee/1940 strain, the mouse-adapted A/California/07/2009-MA (H1N1)pdm09 and A/California/1/1966-MA viruses, and the avian A/ck/Kurgan/05RG/2005 (H5N1) virus with genetically modified low pathogenic HA cleavage site were obtained from the virus collection of the Smorodintsev Research Institute of Influenza (Saint Petersburg, Russia).

All strains of influenza A or B viruses were propagated in 10-day-old chicken embryos at 34 °C or 32 °C, respectively. For ELISA and T-cell stimulation, viruses were purified from infected allantoic fluid by gradient ultracentrifugation through a 30% sucrose cushion at 110,000× *g* for 3 h in a SW28 rotor (Beckman Coulter Inc., CA, USA). Protein content in the purified virus concentrates was measured using the QuDye^®^ Protein Quantification Kit (Lumiprobe, Moscow, Russia) and a Qubit fluorimeter (Thermo Fisher Scientific, MA, USA).

### 2.3. Inactivated Influenza Vaccines

The inactivated influenza vaccine (IIV) component B/Austria/1359417/2021 was provided by Saint Petersburg Scientific Research Institute of Vaccines and Sera (SPbSRIVS, Saint Petersburg, Russia). The B/Austria IIV component contained split virus at a standard 15 μg dose of hemagglutinin (HA) per 0.5 mL. The trivalent inactivated influenza vaccine (TIV) represented a licensed split inactivated vaccine, approved for use in the Russian Federation. A single TIV dose contained 15 μg of HA of each influenza strain recommended by WHO for the 2023–2024 season, Northern hemisphere: A/Victoria/4897/22 (H1N1pdm09), A/Darwin/9/2021 (H3N2), B/Austria/1359417/2021, and B/Phuket/3073/2013.

### 2.4. Animals

Female C57BL/6 and BALB/c mice (6–8 weeks old) were obtained from either the Stolbovaya breeding facility (Moscow region, Russia) for safety and protection studies, or the Nursery for laboratory animals of the Shemyakin–Ovchinnikov Institute of Bioorganic Chemistry RAS (Pushchino, Russia) for immunogenicity and protection studies. The animals were quarantined for a week before the study began. Healthy animals whose body weight differed from the average by no more than 10% were included in the study. Animals were randomly assigned to the experimental and control groups according to the established standard operation procedure. Group allocation was not blinded at any stage of the experiment. Mice that lost more than 35% of their initial body weight in the challenge experiments were humanely euthanized, and the corresponding day was considered the day of the animal’s death. All experiments were conducted in accordance with the European Convention for the Protection of Vertebrate Animals used for Experimental and other Scientific Purposes (ETS No. 123) and were approved by the institutional bioethics committee of the Smorodintsev Research Institute of Influenza (protocols #24 dated 29 November 2023, #35 dated 18 November 2023, #08 dated 3 April 2024).

### 2.5. Safety Study

BALB/c mice (*n* = 18 per group) were infected intranasally with the recombinant B/Lee/NS1ΔC strain or the wild-type B/Lee/40 virus at a dose of 6 lgEID_50_ in 35 µL. Body-weight loss and survival were monitored for 14 days. On days 3 and 5 after infection, four animals from each group were euthanized, and their lungs and nasal turbinates were collected to estimate viral load using the TCID_50_ assay in MDCK cells.

### 2.6. Protection Against the Influenza B/Victoria Virus by the Monovalent Formulation

The protection study was conducted in BALB/c mice (*n* = 20 per group). On day 0, the first group was immunized with the recombinant B/Lee/NS1ΔC virus at a dose of 10^6^ EID_50_ in 35 μL, administered intranasally under light ether anesthesia. The second group was immunized intraperitoneally with the monovalent B/Austria IIV containing 15 μg of HA in 0.5 mL per animal. The B/Austria IIV was administered twice with a two-week interval (on day-14 and day 0). The third group was mock-immunized and served as the infection control group. Three weeks after the last immunization, mice were challenged intranasally with B/Washington/02/2019 at a dose of 2 MLD_50_, 50 μL per animal. The animals were then monitored for 14 days for body-weight loss and survival. On days 3 and 5 after infection, five mice per group were euthanized, and their lungs and nasal turbinates were collected to assess the viral load using the TCID_50_ assay in MDCK cells. On day 7 post-infection, another five mice per group were sacrificed, and their lungs were collected for histological examination.

### 2.7. Immunogenicity of the Monovalent Formulation and Protection Against the Yamagata-Lineage Influenza B Virus

The study was conducted in C57BL/6 mice (*n* = 30 per group). The immunization protocol was the same as described above for the B/Victoria protection study. To evaluate the cellular immune response after vaccination, five mice from each group were euthanized on day 10, and the lungs were collected for lymphocyte isolation and ICS assay. Three weeks after the last immunization, mice were intranasally infected with the B/Phuket/3073/2013 virus at a dose of 6.5 lg TCID_50_ in a volume of 50 µL. On days 3 and 5 after infection, five mice from each group were sacrificed, and the lungs and nasal turbinates were collected to assess the viral load using the TCID_50_ assay. On days 4 and 6 after infection, lungs and spleens were collected from another five animals per group to assess innate and adaptive cellular immune responses. On day 7 post-infection, another five mice per group were sacrificed, and their lungs were collected for histological examination.

### 2.8. Protection Against Heterologous Influenza A and B Viruses by the Trivalent Formulation

The study was conducted in BALB/c mice (*n* = 10 per group/challenge virus). The first group was intranasally immunized with the prototype Flu/NS1ΔC vaccine composition, containing 10^5^ EID_50_ of each NS1ΔC virus strain (A/H1N1, A/H3N2, and B) in a volume of 35 µL. The second group was immunized twice intraperitoneally with the TIV containing 15 µg of HA of each component (A/H1N1pdm09, A/H3N2, and B). The third group was mock-immunized and used as the infection control.

Three weeks after the last immunization, mice were intranasally infected with challenge viruses in a volume of 50 μL per animal under light ether anesthesia. The infectious doses for the mouse-adapted strains A/California/07/2009-MA (H1N1)pdm09 and A/California/1/1966-MA (H2N2) were 10 MLD_50_ and 5 MLD_50_, respectively. The infectious dose for the B/Washington/1/2019 (Victoria-lineage) virus was 5 MLD_50_. As infection with the A/ck/Kurgan/05RG/2005 (H5N1) virus was non-lethal, mice were challenged with the maximum achievable infectious dose of 7.2 lg EID_50_ per animal. Body-weight loss and survival were monitored for 14 days. In case of A/H5N1 infection, which unexpectedly turned out to be sublethal during the virus dose estimation, the endpoint measure of protection was changed from survival to the viral load, mice were euthanized to measure the viral titer in lungs and nasal turbinates on days 3 and 5, and the observation period was only 5 days. In case of B/Victoria additional four mice per group (total *n* = 14 per group) were used to measure the viral load on day 4 post-infection.

### 2.9. Hemagglutination Inhibition Assay (HAI)

Blood serum samples were treated with a Receptor Destroying Enzyme (Denka Seiken, Tokio, Japan) according to the manufacturer’s instructions. To remove nonspecific agglutinins, the sera were additionally treated with an equal volume of 10% chicken red blood cells. Serial twofold dilutions of the sera were prepared in 96-well U-bottom plates using 25 μL of 0.9% NaCl solution. A total of 25 μL/well of the viral antigen containing 4 hemagglutination units was added and incubated for an hour. Then, 50 μL of 0.5% chicken red blood cells were added and incubated for an hour. Antibody titer was estimated as the highest serum dilution that inhibited erythrocyte agglutination.

### 2.10. Real-Time PCR

RNA was extracted from clarified organ homogenates using the MagnoSorb kit 3 (Rospotrebnadzor Central Research Institute of Epidemiology, Moscow, Russia) according to the manufacturer’s instructions. Real-time one-step RT-PCR was performed using the BioMaster RT-qPCR (2×) kit (Biolabmix, Novosibirsk, Russia) following the manufacturer’s protocol. Viral RNA was detected using CDC primers and probes targeting the influenza B NS gene, as described in [20].

### 2.11. Estimation of Virus Infectious Activity—TCID_50_ Assay

Mouse lungs and nasal turbinates were homogenized using a TissueLyser II bead homogenizer (Qiagen, Hilden, Germany). Virus replication activity was assessed by titrating clarified lung and nasal turbinate homogenates in MDCK cell culture. The assay was performed in 96-well culture plates. Cells were infected with 100 µL of 10-fold serial dilutions of the virus-containing material (4 wells per dilution) and incubated for 5 days at 34 °C and 5% CO_2_. Viral replication was determined by hemagglutination of 0.5% chicken erythrocytes in the culture media. The 50% tissue culture infectious dose (TCID_50_) was calculated using the Reed and Muench method [21]. The viral titers were expressed as lg TCID_50_/mL.

### 2.12. Histopathology

Lung tissue was fixed in 10% neutral buffered formalin for 24 h, embedded in paraffin, sectioned at 3–5 µm, and stained with hematoxylin and eosin following standard histological methods. Stained sections were evaluated under a Carl Zeiss AxioSkop 2 plus microscope (Carl Zeiss, Germany). Lung tissue compartments (bronchioli, blood vessels, interstitium, and alveoli) were assessed for the severity of inflammation and focal accumulation of infiltrating cells using a 5-point scale. The resulting score for each animal represented the sum of points for all compartments. Section photographs were captured with an AxioCam ERc5s camera and the AxioVision Rel. 4.8 software (Zeiss, Germany).

### 2.13. Isolation and Stimulation of Lung and Spleen Cell Cultures

To assess cellular immunity, mice were euthanized by cervical dislocation, and the lungs and spleens were aseptically collected. The lungs were perfused with 10 mL of cold DPBS (Biolot, Russia) via the right ventricle, mechanically dissociated, and digested in a 0.5 mg/mL collagenase solution (Sigma, USA) with 10 µg/mL DNase I (Sigma, USA) for 30 min at 37 °C. Spleens were homogenized using a pestle homogenizer. The resulting cell suspensions from both tissues were filtered through a 70 µm cell strainer. The cells were then washed once with DPBS supplemented with 2% fetal bovine serum (FBS, Thermo Fisher Scientific, CA, USA) and 1% penicillin–streptomycin (Biolot, Russia), followed by centrifugation (500× *g* for 7 min at 10 °C). Erythrocytes were lysed using RBC Lysis Buffer (BioLegend, CA, USA), and the remaining cells were washed with DPBS containing 2% FBS. Viable cells were counted on a CytoFLEX flow cytometer (Beckman Coulter Inc., CA, USA) using Zombie Aqua™ viability dye (BioLegend, USA). Subsequently, cells were seeded into flat-bottom 96-well culture plates at a density of 1 × 10^6^ cells per well in 100 µL of RPMI-1640 medium supplemented with 10% FBS and 1% penicillin–streptomycin.

To evaluate antigen-specific T-cell responses, cells were stimulated for 6 h with influenza split vaccine monocomponents (5 µg/well), kindly provided by SPbSRIVS (Saint Petersburg, Russia), or for 24 h with purified viruses (1 µg/well) at 37 °C, 5% CO_2_, in the presence of brefeldin A and anti-CD28 antibodies (both from BioLegend, USA). Unstimulated controls were treated with culture medium containing the same concentrations of brefeldin A and co-stimulatory antibodies.

### 2.14. Flow Cytometry

Innate immune cells were phenotyped using a panel of fluorescently labeled antibodies: CD11b-PE/Cy7, CD11c-PE, MHCII-Alexa Fluor 488, Ly6G-PerCP-Cy5.5, Ly6C-Alexa Fluor 700, CD103-BV605, CD45-APC/Cy7, CD64-BV421, and CD24-BV510 (all from BioLegend, USA). The following cell populations were identified based on standard surface marker expression: neutrophils (SSC^hi^CD45^+^Ly6G^+^), macrophages (CD45^+^MHCII^+^CD64^+^CD11c/CD11b^+^), monocytes (CD45^+^MHCII^−^CD64^+^CD11c/CD11b^+^), and dendritic cells (CD45^+^MHCII^+^CD64^−^CD24^+^CD11c/CD11b^+^CD103^+^/CD103^−^).

To quantify cytokine-producing CD4^+^ and CD8^+^ T lymphocytes following stimulation, cells were stained for surface and intracellular markers. First, cells were incubated with True Stain reagent (BioLegend, USA) to block non-specific Fc receptor-mediated binding. Viability was assessed using Zombie Red™ dye (BioLegend, USA). Surface staining was performed using antibodies against CD8 (PE/Cy7), CD4 (PerCP/Cy5.5), CD44 (BV510), and CD62L (APC/Cy7). Cells were then fixed, permeabilized using the BD Cytofix/Cytoperm™ Kit (BD Biosciences, NJ, USA), and stained intracellularly for cytokines IFN-γ (FITC), TNF-α (BV421), IL-10 (PE/Dazzle™ 594), and IL-2 (PE). All antibodies were from BioLegend, NJ, USA.

Data were acquired on a CytoFlex flow cytometer (Beckman Coulter, USA) and analyzed using the Kaluza Analysis v2.1 software (Beckman Coulter, USA). The gating strategies and representative plots are shown in Supplementary Figures S1 and S2. For ICS analysis, background cytokine levels from non-stimulated cells were subtracted from the corresponding stimulated samples to determine the antigen-specific response.

### 2.15. Statistical Analyses

Primary data were analyzed using GraphPad Prism v10.4.0. The following statistical measures were used to present the data: geometric mean, standard deviation (SD), arithmetic mean, and standard error of the mean (SEM). Differences between group means were assessed using one- or two-way ANOVA, assuming the normal distribution of the data (random distribution of the error) due to the use of linear mice and standard experimental methods. If significant, post hoc Tukey or Dunn tests were applied. Differences were considered statistically significant at *p* < 0.05.

## 3. Results

### 3.1. Generation and Safety Profile of Influenza B Virus with a Truncated NS1 Protein

We created a recombinant influenza B virus with a C-terminal deletion in the NS1 protein based on the high-yield B/Lee/40 strain. Due to a spontaneous mutation in addition to the genetically engineered NS1 (183-range) deletion, we obtained the NS1_1–176_ variant. This virus, B/Lee/NS1ΔC, was passaged several times in chicken embryos, which increased its infectious activity up to 8 lg EID_50_/mL. In vitro experiments demonstrated that B/Lee/NS1ΔC has the features of viruses with a truncated NS1, such as a ts phenotype in chicken embryos and an increased ability to stimulate cytokine response in interferon-competent Calu-3 cells upon infection (Supplementary Figure S2).

**Figure 1:**
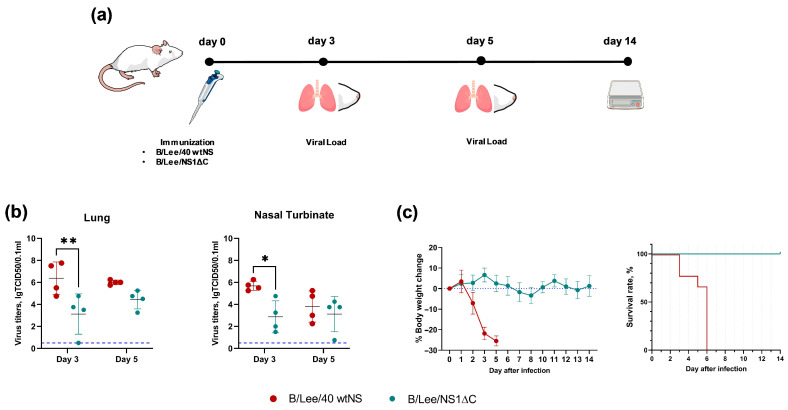
Attenuation of influenza B virus with a truncated NS1 protein in mice. (**a**) Experimental design. (**b**) Viral load in the lungs and the nasal turbinates of infected mice. The blue dotted line marks the detection limit. (**c**) Body-weight dynamics and survival rates in infected mice. Data were analyzed using two-way ANOVA, followed by Tukey’s multiple comparison test (* *p* < 0.05, ** *p* < 0.01).

To confirm that the new B/Lee/NS1ΔC influenza B virus maintains the attenuated phenotype in vivo, we compared it with the wild-type B/Lee/40 virus carrying the full-length NS1 protein, assessing their replication and pathogenicity in mice after intranasal administration. On days 3 and 5 post-infection, lungs and nasal turbinates of infected mice were collected to assess viral load in the respiratory tract. B/Lee/NS1ΔC viral replication was lower than that of the wild-type B/Lee/40 virus. A statistically significant difference in mean viral loads was observed at day 3 post-infection (Figure 1b). The wild-type B/Lee/40 virus caused severe body-weight loss, leading to 100% mortality by day 6 post-infection (Figure 1c). In contrast, B/Lee/NS1ΔC caused no body-weight loss or mortality, indicating that the influenza B virus with a truncated NS1 protein was safe in mice.

### 3.2. Immunogenicity of Influenza B Virus with a Truncated NS1 and Protection Against Influenza B/Yamagata Infection

**Figure 2:**
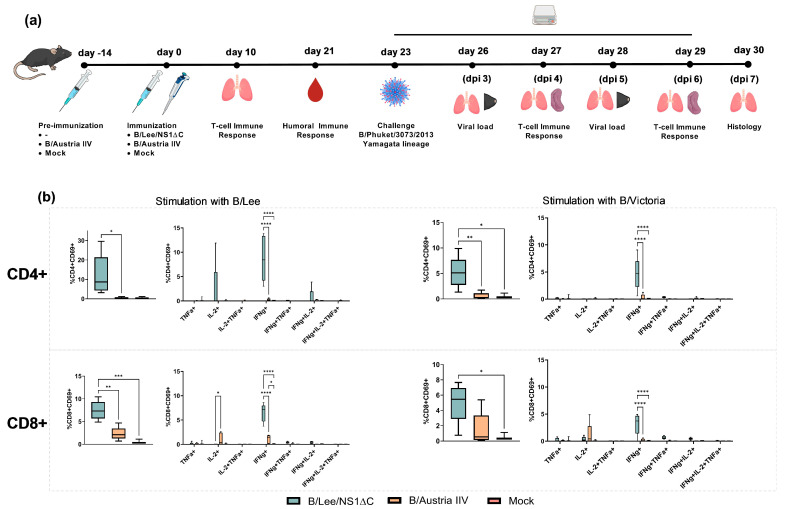
Immunogenicity and protective efficacy of B/Lee/NS1ΔC immunization in C57BL/6 mice. (**a**) Experimental design. (**b**) T-cell response in the lungs of immunized mice at 10 dpi: frequencies of cytokine-producing CD4^+^ and CD8^+^ tissue-resident memory T cells (Trm) in response to stimulation with purified B/Lee or B/Victoria viruses. The left panel shows the total percentage of cytokine^+^ Trm cells, while the right panel shows the percentage of cells producing any combination of IFN-γ, IL-2, and TNF-α. The order of the groups in the legend corresponds to the order of the columns on the graph. Data are presented as box-and-whisker plots (min to max, with the median indicated). Data were analyzed using one- or two-way ANOVA, followed by Tukey’s multiple comparison test (* *p* < 0.05, ** *p* < 0.01, *** *p* < 0.001, **** *p* < 0.0001).

The next step was to assess the immunogenicity of the B/Lee/NS1ΔC virus and investigate whether immunization with B/Lee/NS1ΔC could provide protection against heterologous virus challenge, as shown earlier for influenza A viruses with truncated NS1 [18]. The experimental design is presented in Figure 2a. C57BL/6 mice were immunized intranasally once with 10^6^ EID_50_ of B/Lee/NS1ΔC. The control group was immunized intraperitoneally twice with the B/Austria IIV at a dose of 15 µg HA per mouse. Cellular and humoral immune responses were evaluated on days 10 and 21 post-vaccination, respectively. Three weeks after the last immunization, mice were intranasally infected with 1 MLD_50_ of the B/Phuket/3073/2013 strain. We chose to use a sublethal infection in order to track the dynamics of the immune response during the challenge period.

As expected, immunization with the B/Lee/NS1ΔC virus induced B/Lee/40-specific antihemagglutinating antibodies (GMT = 51), and immunization with B/Austria IIV induced B/Austria/1359417/2021 (Victoria-lineage)-specific antibodies (GMT = 307) with no cross-reactivity to each other or the B/Phuket/3073/2013 Yamagata-lineage virus (Supplementary Figure S4). At the same time, immunization with B/Lee/ΔNS1 led to a pronounced antigen-specific T-cell response to both the B/Lee/40 and B/Malaysia/2506/2004 (Victoria-lineage) viruses, which was characterized by the predominance of monofunctional CD4^+^ and CD8^+^ Trm cells expressing IFNγ (Figure 2b). In the B/Austria IIV group (Figure 2b), cytokine production by tissue-resident T cells was similar to that in the control group, regardless of the stimulation method (B/Lee/40 or B/Malaysia/2506/2004, Victoria-lineage).

**Figure 3:**
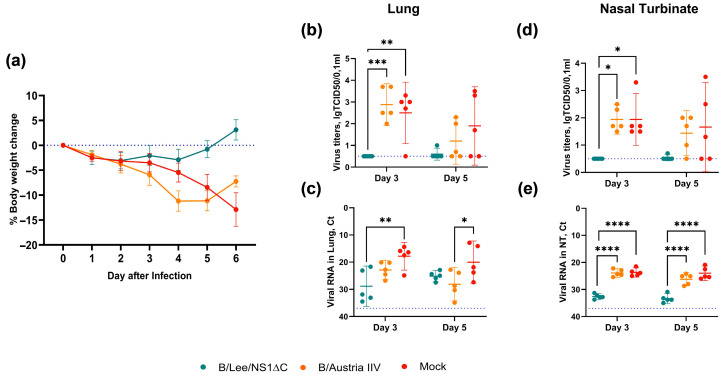
Protective efficacy of B/Lee/NS1ΔC immunization against B/Yamagata-lineage virus infection. (**a**) Body weight dynamics in challenged mice over 7 days after infection. (**b**,**d**) Infectious virus titers in the lungs and nasal turbinates (correspondingly) of challenged mice at 3 and 5 dpi. (**c**,**e**) Virus RNA in the lungs and nasal turbinates (correspondingly) of challenged mice at 3 and 5 dpi. The blue dotted line marks the detection limit. Data were analyzed using two-way ANOVA, followed by Tukey’s multiple comparison test (* *p* < 0.05, ** *p*< 0.01, *** *p* < 0.001, **** *p* < 0.0001).

Subsequent sublethal infection with the heterologous B/Phuket/3073/2013 (Yamagata-lineage) virus led to a significant body-weight loss of up to 13% in the mock-immunized control group and up to 11% in the B/Austria IIV group (Figure 3a). The animals vaccinated with B/Lee/NS1ΔC had lost no more than 3% of their initial weight throughout the experiment and started to gain weight from day 4. In the B/Lee/NS1ΔC mice, the challenge virus replication was almost undetectable in both the lungs and nasal turbinates (Figure 3b,d). For the B/Austria IIV and mock immunized groups, infectious virus was present in the lungs and nasal turbinates of all animals at day 3 post-infection and in 40–80% of animals at day 5. Although RNA of the challenge virus was detected in all animals at both time points, its level was much lower in the B/Lee/NS1ΔC group than in the mock and B/Austria IIV groups (Figure 3c,e).

**Figure 4:**
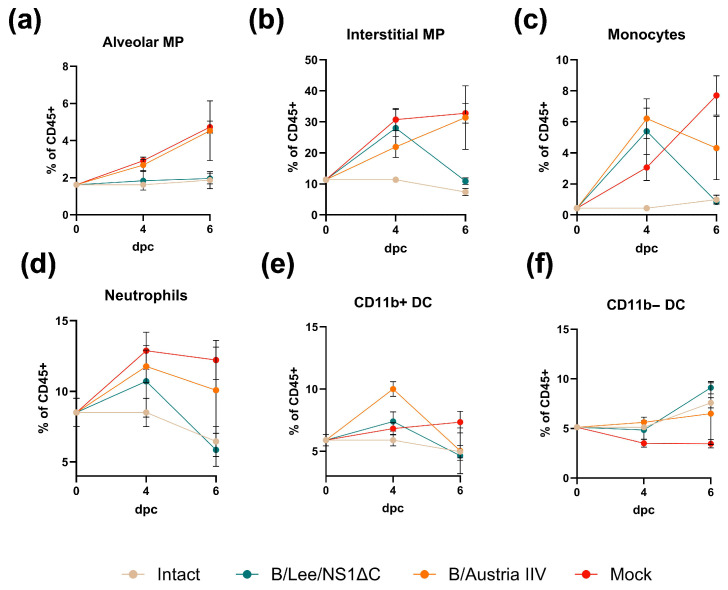
Dynamics of innate immune cell frequencies in the mouse lungs following B/Yamagata virus challenge. (**a**) Alveolar macrophages. (**b**) Interstitial macrophages. (**c**) Monocytes. (**d**) Neutrophils. (**e**) CD11b^+^ dendritic cells. (**f**) CD11b^−^ dendritic cells. Mean and SD values for the frequencies of specific populations are shown. The mean values of the intact group were set as a baseline.

The analysis of innate immune cell dynamics in the lungs during challenge revealed the most pronounced monocyte and neutrophil infiltration in the mock group. This group exhibited a progressive increase in the relative numbers of alveolar macrophages (Figure 4a), interstitial macrophages (Figure 4b), monocytes (Figure 4c), and neutrophils (Figure 4d), alongside a reduction in CD11b^−^ dendritic cells (DCs; Figure 4f).

Although immunization with B/Austria IIV did not prevent inflammatory infiltration overall, it stimulated an influx of CD11b^+^ DCs at day 4 post-infection (Figure 4e). By contrast, the inflammatory response in animals immunized with B/Lee/NS1ΔC was short-lived. The elevated levels of interstitial macrophages, monocytes, and neutrophils observed on day 4 had returned to baseline (naive) levels by day 6 and were significantly lower than those in both the mock and B/Austria IIV groups (Figure 4). Confirming these differences, histological examination of lung tissue at day 7 post-infection revealed a statistically significant decrease in total damage, acute inflammation, and lymphocyte infiltration in mice immunized with B/Lee/NS1ΔC compared to the mock and B/Austria IIV groups (Supplementary Figure S5).

**Figure 5:**
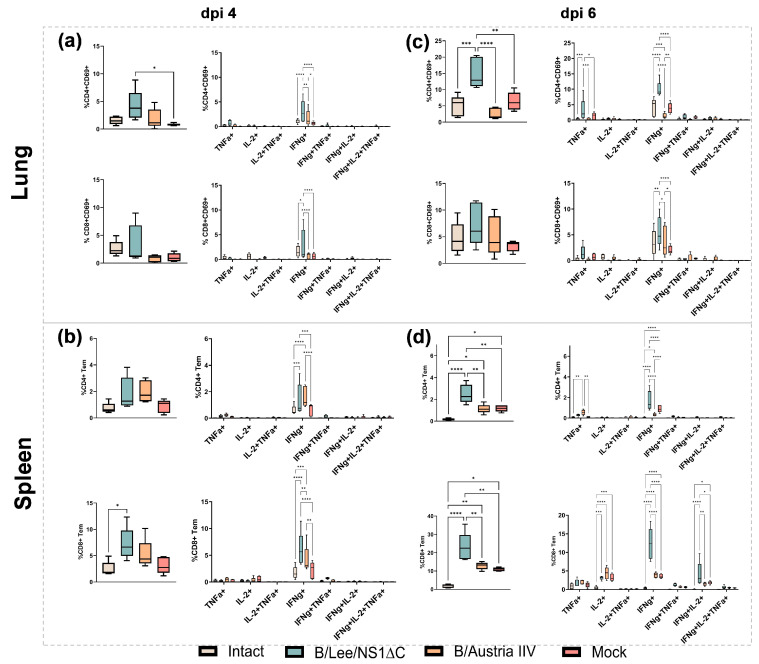
Frequencies of CD4^+^ and CD8^+^ memory T lymphocytes in C57BL/6 mouse lung tissue and spleen at days 4 and 6 post-infection. (**a**,**c**) Antigen-specific CD4^+^ and CD8^+^ Trm responses in the lungs of mice at days 4 and 6 post-infection. (**b**,**d**) Antigen-specific CD4^+^ and CD8^+^ Trm responses in the spleens of mice at days 4 and 6 post-infection. Data were considered statistically significant at *p* < 0.05, as determined by one- or two-way ANOVA followed by Tukey’s multiple comparison test (* *p* < 0.05, ** *p* < 0.01, *** *p* < 0.001, **** *p* < 0.0001).

Only mice vaccinated with B/Lee/NS1ΔC developed the resident memory T cell (Trm) response against the B/Phuket/3073/2013 challenge virus in the lungs. This was characterized by a predominance of monofunctional CD4^+^ and CD8^+^ Trm cells expressing IFNγ (Figure 5a,c). In the spleen, the CD4^+^ effector memory T cell (Tem) response specific to B/Phuket/3073/2013 was detected in all challenged groups. However, the B/Lee/NS1ΔC group showed a more pronounced response, dominated by monofunctional IFNγ^+^ CD4^+^ Tem cells as well (Figure 5b,d). The systemic CD8^+^ Tem response was also significantly stronger in the B/Lee/NS1ΔC group than in the B/Austria IIV and mock groups. This enhanced response involved not only a further increase in cytokine production by day 6 post-infection, but also a broader functionality of the responding T-cell populations (Figure 5b,d).

Overall, a single intranasal vaccination with B/Lee/NS1ΔC stimulated a cross-reactive CD4^+^/CD8^+^ Trm response in the lungs, indicating protective activity against heterologous influenza B/Yamagata infection. Immunization with the B/Lee/NS1ΔC strain limited the inflammatory response in the lungs during infection, which correlated with the pathological changes reflected by weight-loss dynamics.

### 3.3. Protective Efficacy of Influenza B Virus with a Truncated NS1 Protein Against B/Victoria Challenge

**Figure 6:**
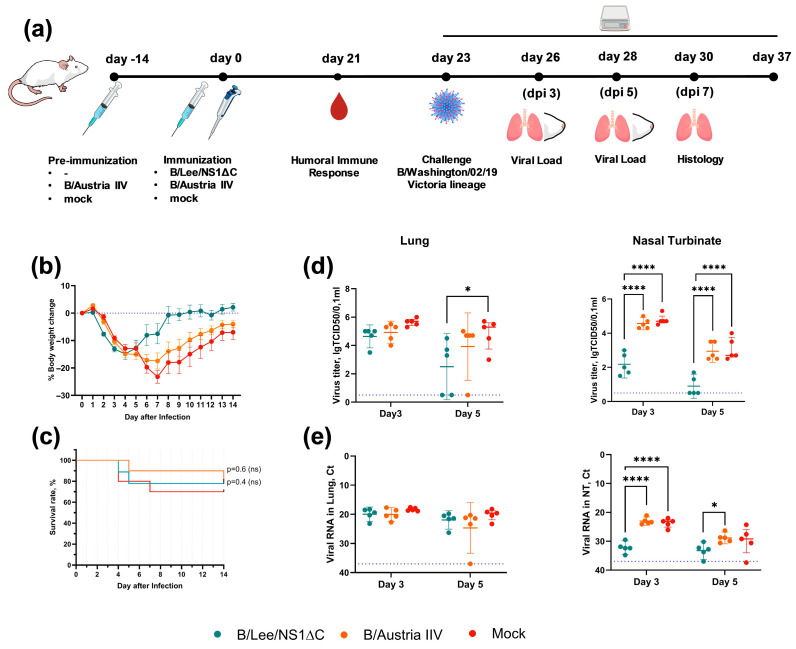
Protective efficacy of B/Lee/NS1ΔC immunization against B/Victoria-lineage virus infection. (**a**) Experimental design. (**b**) Body weight dynamics during infection. (**c**) Survival rate. Data was analyzed using the Mantel–Cox log-rank test; ns—not significant. (**d**) Infectious titers in the lungs and the nasal turbinates of challenged mice at 3 and 5 dpi. (**e**) Viral RNA in the lungs and nasal turbinates of challenged mice at 3 and 5 dpi. The blue dotted line marks the detection limit. Data were analyzed using two-way ANOVA, followed by Tukey’s multiple comparison test (* *p* < 0.05, **** *p* < 0.0001).

Using a similar experimental design as for the B/Yamagata challenge, we evaluated the protective efficacy of B/Lee/NS1ΔC immunization against influenza B viruses of the Victoria lineage (Figure 6a). BALB/c mice were immunized intranasally once with 10^6^ EID_50_ of the B/Lee/NS1ΔC virus or intraperitoneally twice with the B/Austria IIV at a dose of 15 µg HA per mouse. Three weeks after the last immunization, mice were intranasally infected with 2 MLD_50_ of the B/Washington/02/2019 Victoria-lineage strain. The sublethal dose was chosen to allow monitoring of lung pathology development after infection.

During the challenge, mock-immunized mice and mice that received B/Austria IIV lost up to 25% of their initial body weight by day 7 post-infection. The B/Lee/NS1ΔC group lost only up to 15% of their body weight by day 4 post-infection and began to recover at day 5 (Figure 6b). No significant difference in survival rates was observed between the groups (Figure 6c).

At day 3 post-infection, the viral load in the lungs was similar in all groups. At day 5, viral RNA levels were also the same; however, the infectious viral load in the lungs of the B/Lee/NS1ΔC group was significantly lower than in the mock and B/Austria IIV groups (Figure 6d,e). In the nasal turbinates of mice immunized with B/Lee/NS1ΔC, both viral RNA load and infectious viral titers were lower compared to control and IIV-immunized animals at both time points (Figure 6d,e). Moreover, at day 5, viral replication in the nasal turbinates was observed in only 2 of 5 mice in the B/Lee/NS1ΔC group (Figure 6d).

**Figure 7:**
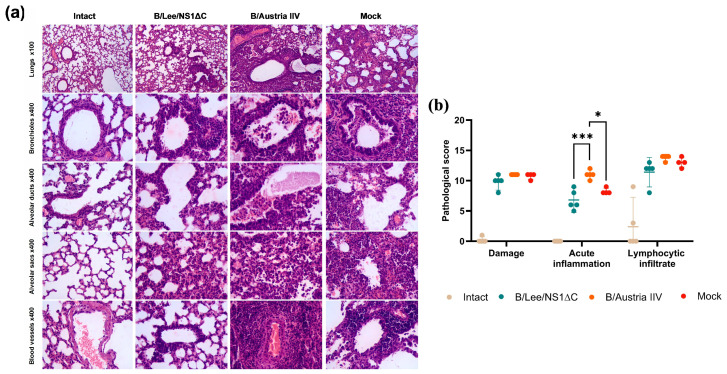
Histopathological changes in the mouse lungs at day 7 post-infection. (**a**) Representative microphotographs of lung sections stained with hematoxylin and eosin, showing the most pronounced pathological changes. (**b**) Histopathological summary score of lung examination. Data were analyzed using two-way ANOVA, followed by post hoc Tukey’s multiple comparison test (* *p* < 0.05, *** *p* < 0.001).

Histopathological analysis at day 7 post-infection revealed that mice immunized with B/Lee/NS1ΔC developed minimal or mild inflammatory signs in the lungs, with focal epithelial injury and peribronchial infiltration by lymphocytes and macrophages. The B/Austria IIV group showed obstructive bronchitis and bronchiolitis with moderate interstitial inflammation, epithelial damage, and mild perivascular infiltration. In the mock group, we observed severe lung damage with obstructive bronchitis, bronchiolitis, interstitial inflammation, epithelial necrosis, and moderate perivascular infiltration (Figure 7a). Semi-quantitative assessment showed that B/Lee/NS1ΔC immunization significantly reduced the acute inflammatory process in the lungs during infection, primarily in the bronchioles and alveoli (Figure 7b).

Overall, our data indicate that immunization with B/Lee/NS1ΔC provided protection against B/Victoria-lineage challenge by reducing viral load in the respiratory tract and attenuating acute lung inflammation during infection.

### 3.4. Cross-Protection by a Trivalent Formulation of NS1ΔC Influenza Viruses Against Divergent Influenza A and B Strains

**Figure 8:**
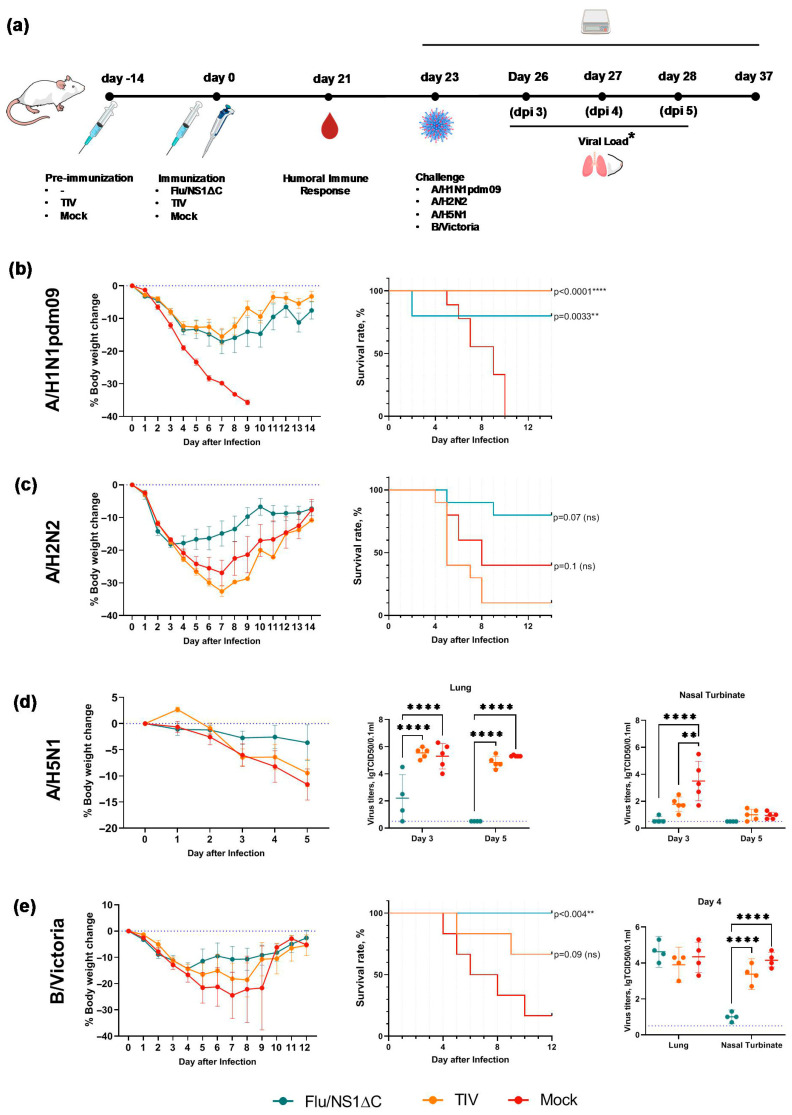
Protective efficacy of immunization with the trivalent composition of NS1ΔC influenza viruses against influenza A and B viruses. (**a**) Experimental design * Viral load was estimated only after A/H5N1 and B/Victoria challenge. (**b**) Body-weight loss dynamics and survival rates in vaccinated animals challenged with A/H1N1pdm09. (**c**) Body-weight loss and survival rates of vaccinated animals challenged with A/H2N2. (**d**) Body-weight loss and viral load in the respiratory tract of vaccinated animals challenged with A/H5N1. The observation period was only 5 days, as animals were euthanized to measure the viral load (**e**) Body-weight loss, survival rates and viral load in the respiratory tract of vaccinated animals challenged with B/Victoria. Survival rates were compared using the Mantel–Cox log-rank test. Viral titers were compared using two-way ANOVA, followed by post hoc Tukey’s multiple comparisons test. ** *p* < 0.01, **** *p* < 0.0001, ns—non significant. The blue dotted line marks the detection limit. Animals, who lost more than 35% of their initial body weight were humanely euthanized, and the corresponding events were counted as animal death.

The final stage of the study was to evaluate whether the observed cross-protective properties of the recombinant influenza A and B viruses with truncated NS1 proteins are affected by potential interference when delivered simultaneously [22,23]. To test this, we immunized mice with the prototype trivalent vaccine Flu/NS1ΔC containing influenza A/H1N1, A/H3N2, and B NS1ΔC viruses in equal doses (10^5^ EID_50_), and then challenged them three weeks later with divergent influenza A and B strains. Mice immunized with the seasonal TIV (containing 15 µg HA of A/H1N1pdm09, A/H3N2, and B/Victoria) and mock-immunized animals were used as controls. The experimental design is shown in Figure 8a.

Mice infected with the mouse-adapted A/H1N1pdm09 strain rapidly lost body weight, with 100% mortality observed in the control mock-immunized group, whereas weight loss in the Flu/NS1ΔC and TIV groups was generally slower. From 7 dpi onward, animals in both immunized groups began to recover from infection, which resulted in significantly higher survival rates. In the trivalent Flu/NS1ΔC group, survival reached 80%, while 100% survival was observed in the TIV group, consistent with the expected protective effect of the inactivated vaccine against infection with a homologous drifted variant strain (Figure 8b).

Infection with the mouse-adapted A/H2N2 virus resulted in a rapid body-weight loss in all experimental groups. Mice in the control group lost up to 30% of their initial body weight; lethal outcomes were observed from day 4 post-infection, and the survival rate in this group was 60%. The survival rate of TIV-immunized animals was even lower and amounted to only 10%, indicating a lack of protection. In the Flu/NS1ΔC group, the survival rate was 80%, and a decrease in body weight was observed only during the first three days after infection, followed by recovery (Figure 8c).

Sublethal infection with the avian influenza A/H5N1 virus led to a 10–12% body-weight loss in the control and TIV groups, while mice immunized with Flu/NS1ΔC lost no more than 4% of their initial body weight during the observation period (Figure 8d). Flu/NS1ΔC also promoted fast viral clearance: at day 3 post-infection, viral load in the lungs was 2.5 times lower in the Flu/NS1ΔC group than in the control and TIV groups, and it fell below the detection limit by day 5. Flu/NS1ΔC also prevented replication of the challenge A/H5N1 virus in the nasal turbinates, as live virus was detected in only one animal at day 3 post-infection. Except for a reduced viral load in the nasal turbinates at day 3, the seasonal TIV did not demonstrate any protective effect against A/H5N1 virus infection (Figure 8d).

During lethal infection with the B/Victoria virus, mortality in the control group was observed starting from day 4 post-infection, with the final survival rate of 33%. TIV immunization expectedly increased the survival rate up to 67%, and no mortality was observed in the Flu/NS1ΔC group (Figure 8e). However, all animals experienced infection, and by day 4 post-infection, body-weight loss was comparable across all groups (around 15%). Starting from day 5, differences in recovery trends were observed: mice in both immunized groups gained weight faster than control animals. On day 12 all survived animals recovered and the observation was stopped. There was no difference in viral load in the lungs at day 4 post-infection, although animals immunized with Flu/NS1ΔC showed a significantly lower viral titer in the nasal turbinates.

Overall, our findings allow us to conclude that combining the three NS1ΔC influenza viruses—A/H1N1, A/H3N2, and B—into a single formulation did not compromise their cross-protective properties, and that a single intranasal immunization with the trivalent Flu/NS1ΔC formulation protected mice against divergent influenza A and B viruses, including the potentially pandemic A/H5N1 and A/H2N2 strains.

## 4. Discussion

Creating a universal influenza vaccine that provides long-lasting protection against a broad spectrum of influenza A and B viruses is still one of the main objectives in global infectious disease control. According to the Universal Influenza Vaccine Technology Landscape, nearly 200 vaccine candidates are currently in preclinical studies, and 44 candidates are advancing through various phases of clinical trials [24]. The main strategies to broaden vaccine protection include redirecting the antibody response to conserved regions of viral proteins; activating cross-protective T-cell responses against internal viral proteins; broadening both B- and T-cell responses using adjuvants, nanoparticles, or virus-like particles (VLPs); and directly stimulating cross-specific mucosal immunity or tissue-resident memory T cells [25].

Vaccines based on influenza viruses with a modified NS1 protein combine three out of the four strategies described above. Removing or truncating the viral NS1 protein—the main antagonist of the interferon immune system—enhances immune response by increasing cytokine synthesis, which gives NS1-modified influenza viruses “self-adjuvanted” properties [12,26]. At the same time, intranasal delivery of NS1-modified influenza viruses ensures complex local immunity in the respiratory tract [8] and induces a strong cross-specific T-cell immune response [10,18], even in aged animals [27]. Moreover, several clinical trials have already demonstrated the safety and immunogenicity of vaccines based on influenza viruses with deleted NS1 [28,29,30,31,32,33] and on the influenza A virus with truncated NS1 (NCT03017378) [34].

The immunological mechanisms underlying cross-protection provided by influenza A viruses with deleted or truncated NS1 are well studied in animal models [10,18,35,36]. In contrast, few studies have explored the universal vaccine potential of influenza B viruses with NS1 deletions of varying lengths, and only a few have been tested and shown to provide protection against heterologous challenge [10,12].

In this study, we generated an influenza B virus with a C-terminal deletion in NS1, based on the structural homology with influenza A NS1_1–124_ [14]. Previously described influenza B viruses with truncated NS1 protein demonstrated poor replication in chicken embryos [13,37], and NS1-deleted influenza B vaccine strains could be produced only in Vero or MDCK cell cultures [10,11]. In contrast, our mutant strain B/Lee/NS1ΔC replicated to up to 8.0 lgEID_50_/mL, sufficient for vaccine production in chicken embryos.

The constructed B/Lee/NS1ΔC virus showed an attenuated phenotype in mice: intranasal administration caused no weight loss or mortality. Similarly to other influenza viruses with truncated NS1 [12,26,38], its replication level in the mouse respiratory tract was significantly lower than that of the wild-type strain. Earlier it was shown, that influenza A viruses with truncated NS1(1-124) also demonstrated some level of replication in mice [26,39]. In comparison to humans, most laboratory mouse strains carry a non-functional Mx1 gene, which limits its comparability to humans [40]. More significantly, in clinical trial of the live influenza A vector with truncated NS1 no live virus was recovered from the nasal swabs of vaccinated volunteers [34], confirming the replication-deficient type of the vaccine. It must nevertheless be acknowledged that Flu/NS1ΔC is not supposed to be used in immunocompromised people.

A single intranasal administration of B/Lee/NS1ΔC was sufficient to generate high titres of HAI antibodies in naïve mice, whereas the inactivated B/Austria formulation required two doses (Supplementary Figure S4). Antibodies to heterologous or drift viruses, including the challenge strain, were under the detection limit for both groups (Figure S4). At the same time, immunization with the B/Lee/NS1ΔC strain induced CD4^+^ and CD8^+^ cells in the lungs, which produced IFNγ in response to stimulation with both homologous and heterologous viruses (Figure 2). This was not observed after immunization with the B/Austria IIV. These findings align with previous reports on cross-specific T-cell response in the lungs generated by influenza A viruses with truncated NS1 and LAIV [18,41].

Next, we confirmed cross-protective properties of the B/Lee/NS1ΔC vaccine strain against infection with two influenza B virus lineages in a sublethal mouse model (Figure 3 and Figure 6). We observed the early activation of resident memory T cells in the lungs of mice immunized with B/Lee/NS1ΔC, which were cross-specific to the challenge virus (Figure 5). Furthermore, we examined the dynamics of the innate immune response to influenza in immunized animals, which affects the progression of infection [9]. We showed that immunization with B/Lee/NS1ΔC limited the inflammatory response in the lungs following infection, as reflected by its rapid decrease to the baseline values between 4 dpi and 6 dpi. Notably, this coincided with the time point when the weight loss dynamics shifted from the decline to recovery in B/Lee/NS1ΔC immunized animals, unlike in IIV or control groups that continued to lose weight and had increased inflammatory cell counts even at 6 dpi. This observation was supported by histopathological examination at 7 dpi, which confirmed lower immune cell infiltration in the lung tissue of B/Lee/NS1ΔC-immunized mice. These results are consistent with earlier findings showing that influenza A viruses with truncated or deleted NS1 can modulate the early immune response during a subsequent infection [18,19,42,43].

In our study, immunization with B/Austria IIV did not protect mice against the sublethal B/Washington challenge. According to the WIC report [44], the antigenic difference between B/Austria and B/Washington viruses, both Victoria lineage, can be considered the vaccine strain mismatch. Therefore, IIV was expected not to prevent infection but rather to decrease disease severity. The lack of a detectable protective effect from IIV could be attributed to the relatively low severity of infection in our model, especially compared with other studies in which mice immunized with antigenically drifted influenza B HA survived a 100% lethal B/Washington infection [45,46]. Indeed, in the trivalent vaccine experiment, the TIV protection effect was observed as expected when a more severe infection was induced. Importantly, the trivalent Flu/NS1ΔC composition also showed better protection in this case. This is especially relevant given that most universal influenza vaccine studies use lethal challenge models, which do not fully reflect real-life scenarios where non-pandemic but drifted viruses emerge in the human population.

The dominance of influenza A virus pandemics supports the common view that influenza B virus causes a milder disease than influenza A virus. Nevertheless, recent surveillance data suggest that influenza B virus disease burden may be comparable or even greater, particularly in young children [47,48,49,50]. Thus, cross-protection against drifted influenza B viruses is no less important than cross-protection against influenza A, and including a B component in universal influenza vaccines is highly desirable. The currently developed universal vaccines target mainly influenza A viruses with the aim to protect against pandemic strains and that lack the influenza B component [51]. Moreover, immunization schemes often contain multiple vaccinations to get the cross-specific response, as in the case of HA-stalk-based and COBRA-based vaccines [52,53,54]. The Flu/NS1ΔC vaccine contains both influenza A and B components and showed cross-specific immune response following single immunization. At the same time, the presence of several components in a vaccine raises the question of their interference. In our cross-protection study of the trivalent Flu/NS1ΔC formulation containing the A/H1N1, A/H3N2, and B NS1ΔC influenza viruses, we found no indication of interference when the NS1ΔC vaccine strains were delivered together, unlike the interference reported for wild-type influenza viruses [22,23]. A single intranasal immunization with the trivalent Flu/NS1ΔC influenza vaccine was enough to ensure protection against divergent strains of influenza, including a drifted B/Victoria strain and potentially pandemic A/H2N2 and A/H5N1 viruses.

## 5. Conclusions

Overall, this study demonstrated that immunization with a recombinant influenza B virus with an NS1 C-terminal effector domain deletion can provide cross-protection mediated by cross-specific T-cellular immune response and innate immunity restraining. The trivalent formulation of influenza A/H1N1, A/H3N2, and B NS1ΔC viruses administered intranasally induced broad cross-protection against infection with distant influenza A and B viruses. The developed trivalent Flu/NS1ΔC influenza vaccine offers a promising approach to improving seasonal influenza vaccination and pandemic preparedness.

## Data Availability

The data are not publicly available due to the institution’s policy, and could be provided upon reasonable request to the corresponding author.

## Supplementary Materials

The following supporting information can be downloaded at: https://www.mdpi.com/article/10.3390/vaccines14010043/s1, Figure S1: Gating strategy to identify adaptive immune cell populations; Figure S2: Gating strategy to identify innate immune cell populations; Figure S3: In vitro reproduction and phenotypic characteristics of the B/Lee/NS1ΔC virus; Figure S4: Hemagglutination inhibition antibody titers in mice immunized with B/Lee/NS1ΔC or B/Austria IIV three weeks after the last immunization; Figure S5: Histopathological changes in C57BL/6 mouse lungs at day 7 post-infection with the B/Phuket virus.

## Abbreviations

The following abbreviations are used in this manuscript:

IIV	Inactivated Influenza Vaccine
HA	Haemagglutinin
LAIV	Live Attenuated Influenza Vaccine
TIV	Trivalent Inactivated Influenza Vaccine
TCID	Tissue Culture Infective Dose
MLD	Mouse Lethal Dose
EID	Egg Infective Dose
ANOVA	Analysis Of Variance
Trm	Resident memory T cell
Tem	Effector memory T cell
