# Supplementary material for: Novel Intranasal Replication-Deficient NS1ΔC Flu Vaccine Confers Protection from Divergent Influenza A and B Viruses in Mice
Source: Vaccines (Basel). 2025 Dec 30;14(1):43. doi: 10.3390/vaccines14010043 (PMC12846425; doi:10.3390/vaccines14010043)
Supplement: Supplementary file 1 [file vaccines-14-00043-s001.zip › Supplementary figures.pdf]

Supplementary figures

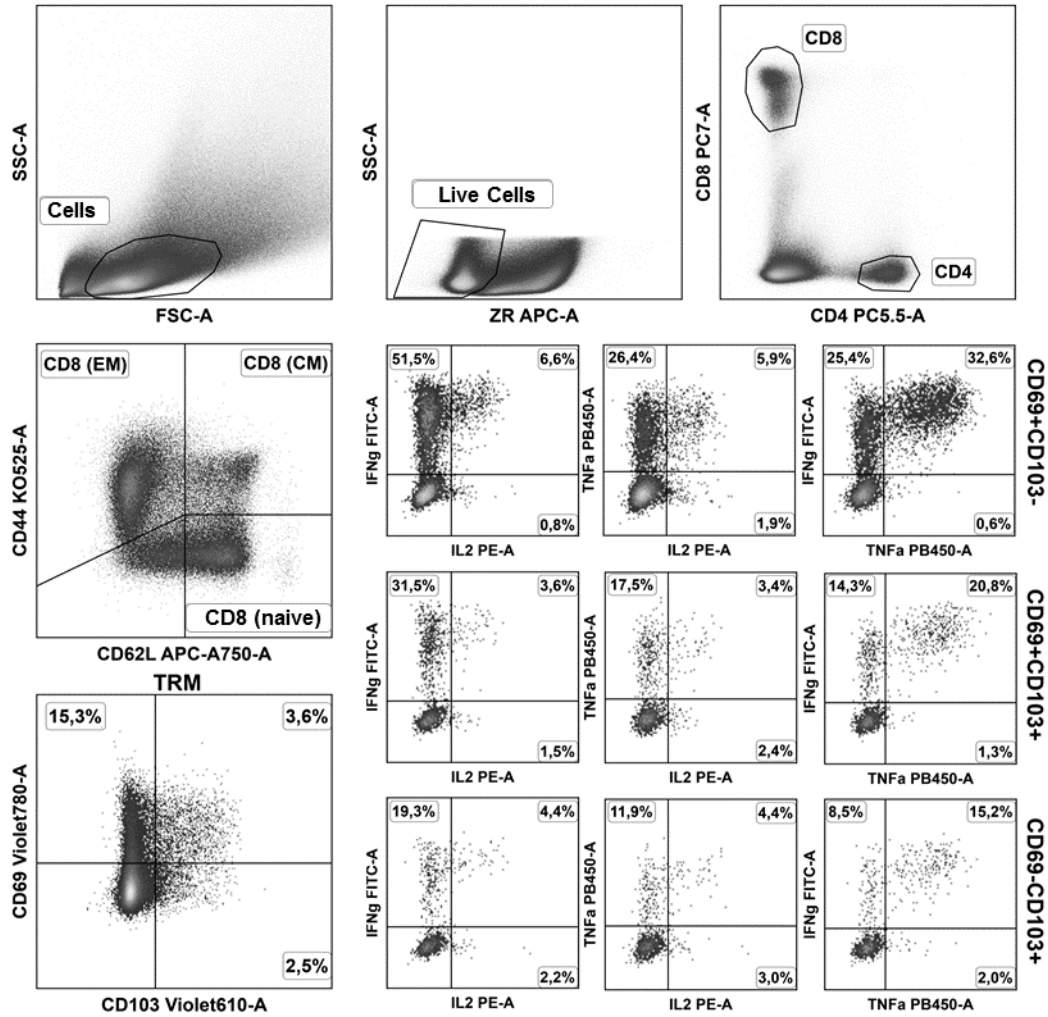

Figure S1. Gating strategy to identify adaptive immune cell populations

Nonviable cells were excluded from the analysis based on the forward and side scatter (FSC/SSC) parameters and Zombie Red viability marker staining. The population of viable T lymphocytes was divided into T helper cells (CD4+) and cytotoxic T cells (CD8+) based on the corresponding CD4 and CD8 surface markers. According to the expression levels of the CD44 and CD62L markers, subpopulations of naive T lymphocytes (CD44-CD62L+), central (Tcm: CD44+CD62L+), and effector (Tem: CD44+CD62L-) memory T cells were distinguished. Tissue-resident memory T cells (Trm) were differentiated from effector memory T cells (CD44+CD62L-) based on increased expression of CD69 and/or CD103. The Trm population includes cells with the CD69+CD103- and CD69+CD103+ phenotypes. Subpopulations of activated tissue-resident CD8+ memory T cells were then assessed based on their expression of intracellular cytokines IFNγ, TNFα, and IL2.

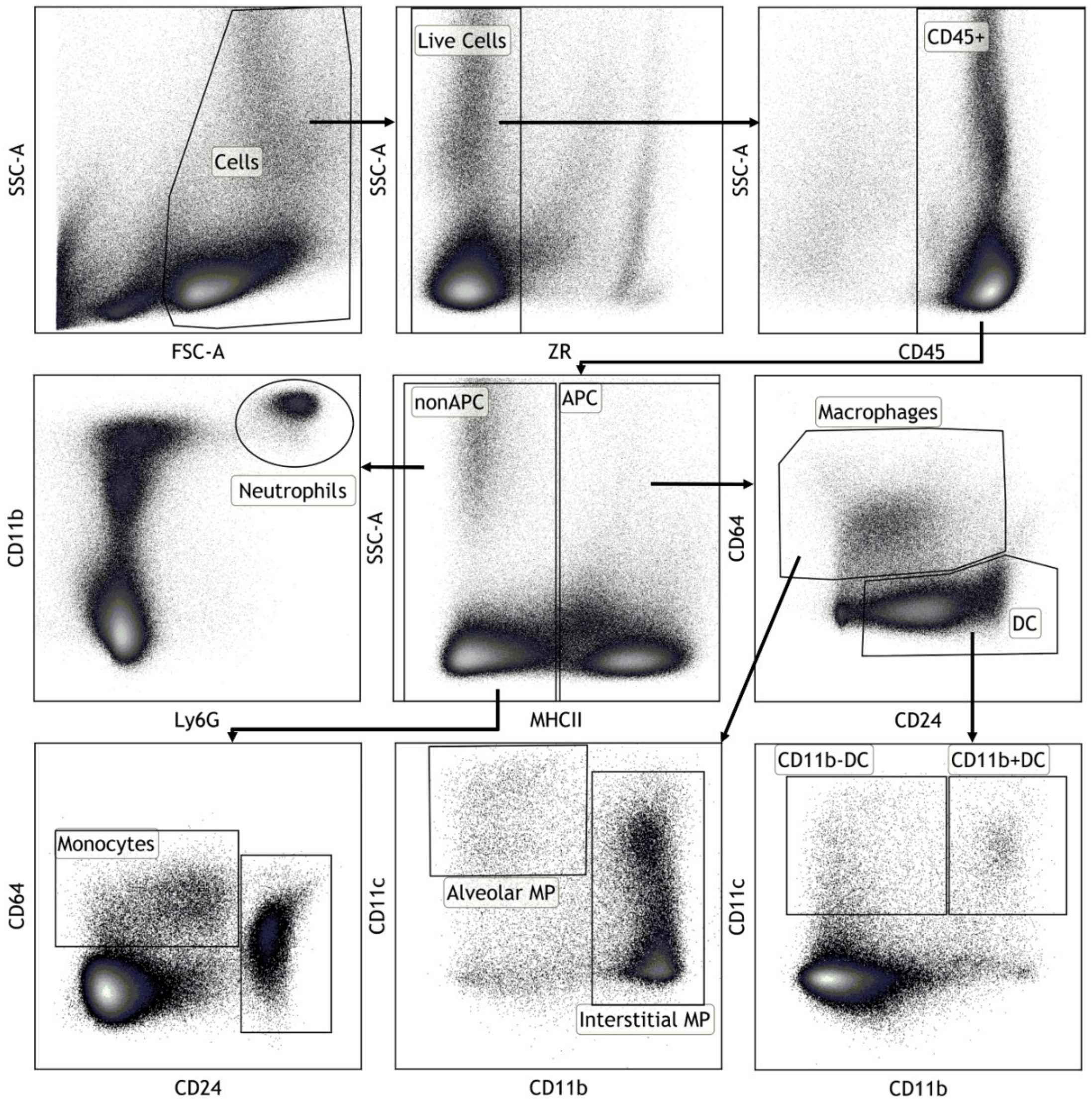

Figure S2. Gating strategy to identify innate immune cell populations.

After excluding doublets by FSC-A/FSC-H (not shown in the figure) and isolating the live cell population based on light scattering characteristics (FSC-A/SSC-A -Cells) and Zombie Red dye staining, immunocytes were gated according to the presence of the CD45 marker (CD45+). During further analysis, the following cell populations were distinguished: SSChiCD45+Ly6G+ (Neutrophils); MHCII-CD64+CD24+ (Monocytes); MHCII+CD64+ CD11c+CD11b- (Alveolar MP); MHCII+CD64+CD11b+ CD11c+/- (Interstitial MP); CD45+CD11c+CD11b-MHCII+CD64-CD24+ (CD11b- Dendritic cells) and `CD45+CD11c+CD11b+MHCII+CD64-CD24+ (CD11b+ Dendritic cells).

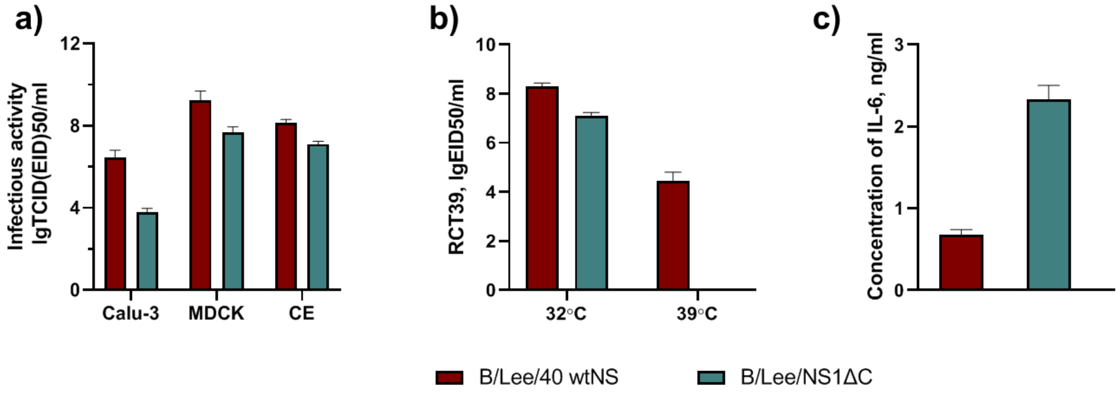

Figure S3. *In vitro* reproduction and phenotypic characteristics of the B/Lee/NS1ΔC virus

(**a**) Infectious activity of the B/Lee/NS1ΔC virus in interferon-competent substrates in comparison to the wild-type virus with full-size NS1. (**b**) Temperature sensitivity in chicken embryo substrate. (c) Cytokine production by Calu-3 cells 18 h after infection with viruses at an moi = 1 $TCID_{50}$/cell.

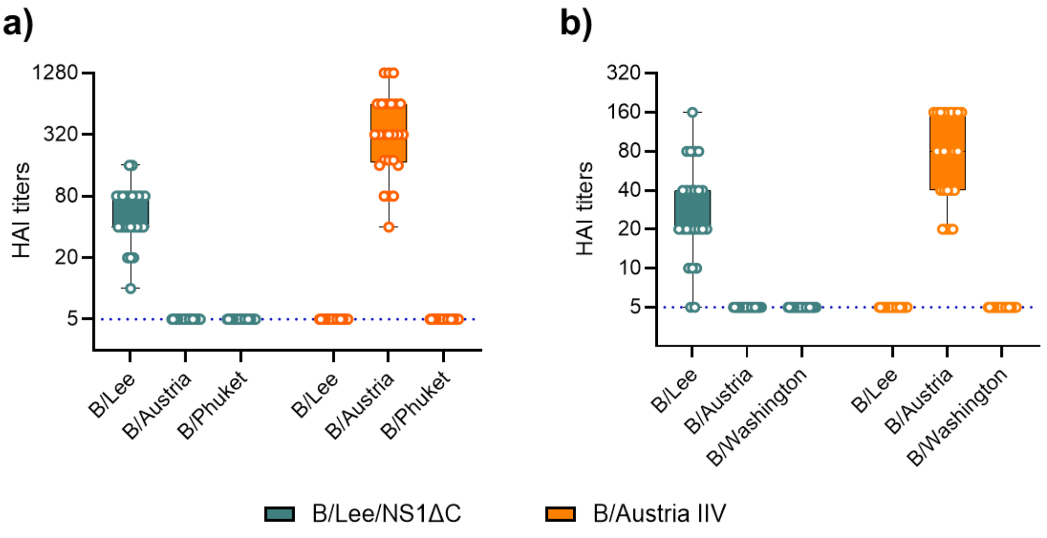

Figure S4. Hemagglutination inhibition antibody titers in mice immunized once with B/Lee/NS1ΔC or twice with B/Austria IIV three weeks after the last immunization (data on antibody response after single B/Austria IIV immunization not shown due to the absence of any detectable antibody titres).

(a) Antibody response in immunized C57BL/6 mice before the B/Phuket (Yamagata-lineage) challenge. **(b)** Antibody response in immunized BALB/c mice before the B/Washington (Victoria-lineage) challenge. Virus antigens used for HAI assay are indicated above the axis. Data presented as box-and-whiskers plots (Min to Max) with all points shown and the median indicated.

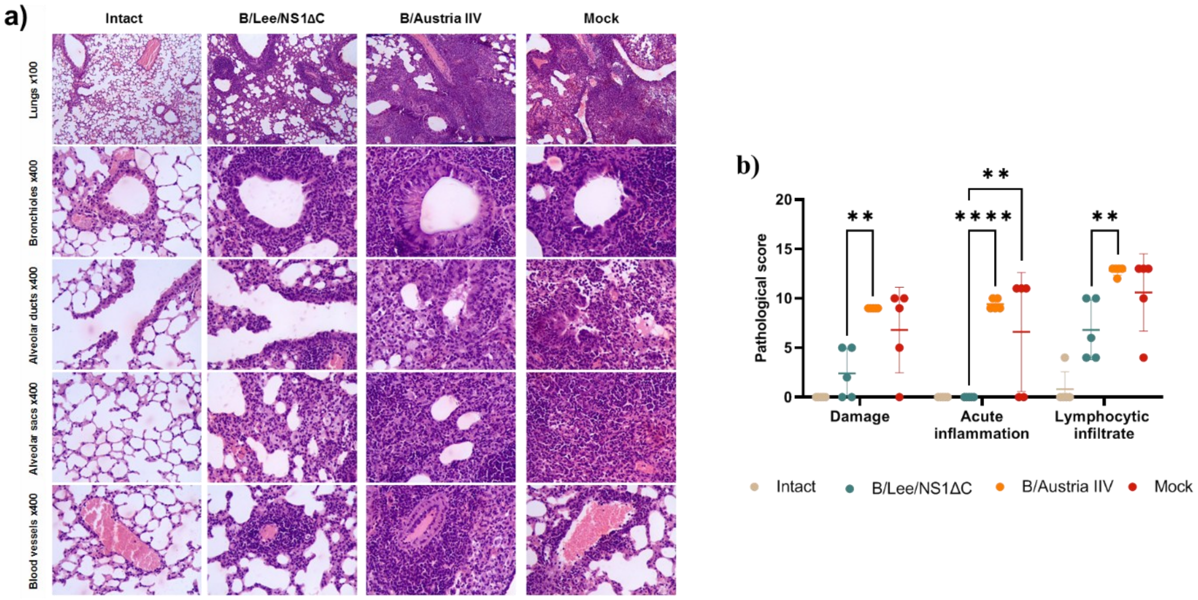

Figure S5. Histopathological changes in C57BL/6 mouse lungs at day 7 post-infection with the B/Phuket virus. **(a)** Representative microphotographs of lung sections stained with hematoxylin and eosin, showing the most pronounced pathological changes. **(b)** Histopathological summary score of lung examination. **p<0.01, ****p<0.0001 calculated using two-way ANOVA followed by post-hoc Tukey test.
